# A cross-sectional study of the prevalence and factors associated with symptoms of perinatal depression and anxiety in Rwanda

**DOI:** 10.1186/s12884-020-2747-z

**Published:** 2020-01-31

**Authors:** Marie Providence Umuziga, Oluyinka Adejumo, Michaela Hynie

**Affiliations:** 1 School of Nursing, College of Medicine and Health Sciences, University of Rwanda, 3286, Kigali, Rwanda; 2 Department of Psychology, York University, 4700 Keele Street, Toronto, Ontario M3J 1P3 Canada

**Keywords:** Perinatal depression, Perinatal anxiety, Maternal mental health, Social support, Rwanda, Social factors of mental health

## Abstract

**Background:**

Perinatal depression and anxiety are increasingly recognized as important public health issues in low and middle-income countries such as Rwanda and may have negative consequences for both mothers and their infants. Maternal mental health may be particularly challenged in Rwanda because of the prevalence of risk factors such as poverty, low education levels, negative life events and marital problems. However, there are limited data about perinatal depression and anxiety symptoms in Rwanda. This study thus aimed to explore the prevalence of symptoms of perinatal depression and anxiety in Rwanda, and factors associated with them.

**Methods:**

A sample of 165 women in the perinatal period (second and third trimester of pregnancy, up to 1 year postnatal) were interviewed individually over 1 month in October 2013. Women were interviewed at 5 of 14 health centres in the Eastern Province or the affiliated district hospital. Participants answered socio-demographic questions and scales measuring symptoms of perinatal depression (EPDS: Edinburgh Postnatal Depression Scale) and anxiety (SAS: Zung Self-rating Anxiety Scale).

**Results:**

Among women in the antenatal period (*N* = 85), 37.6% had symptoms indicating possible depression (EPDS ≥10) and 28.2% had symptoms associated with clinical levels of anxiety (SAS > 45). Among women within the postnatal period (*N* = 77), 63.6% had symptoms of possible depression, whereas 48,1% had symptoms of probable anxiety. Logistic regression showed that symptoms of postnatal depression were higher for respondents who had four or more living children relative to those having their first child (Odds Ratio: 0.07, C.I. = 0.01–0.42), and for those with a poor relationship with their partner (Odds Ratio: .09, C.I. =0.03–0.25). Any lifetime exposure to stressful events was the only predictor of symptoms of postnatal anxiety (Odds Ratio = 0.20, C.I. = 0.09–0.44).

**Conclusions:**

Symptoms of postnatal depression and anxiety were prevalent in this Rwandan sample and most strongly predicted by interpersonal and social factors, suggesting that social interventions may be a successful strategy to protect against maternal mental health problems in the Rwandan context.

## Background

Depression and anxiety occurring in the perinatal period, the time during pregnancy and up to 1 year following birth, can have serious consequences for women’s mental health and for that of their infants [1–6]. Perinatal depression and anxiety are associated with reduced social participation among mothers and maternal sensitivity to their infants with elevated risks of infant malnutrition, increased rates of physical illness and subsequent depressive episodes [7–13].

Perinatal depression and anxiety are referred to as Common Perinatal Mental Disorders (CPMDs) as they represent the most common mental health problems faced by women during the perinatal period [14, 15]. Rates of first onset and severe depression are three times higher in the postnatal period than in other periods of women’s lives, suggesting that this is a particularly vulnerable time [16]. There is also increasing evidence that CPMDs are two to three times more prevalent among pregnant women and mothers of infants in low-income countries (LMICs), including Rwanda, than in high-income countries [17–19].

A systematic review of studies conducted in LMICs reported that the prevalence of CPMDs was 15.6 and 19.8% antenatally and postnatally, respectively [20]. In a recent study by Gelaye and colleagues [21], the prevalence rates of antenatal depression in LMICs ranged between 19 to 25% whereas postnatal depression prevalence was between 10 to 20%. For anxiety, the global estimates are about 14–16% during pregnancy and 8–10% postnatally [19]. On the African continent, prevalence rates vary widely. It has been shown that the estimated prevalence of CPMDs in sub-Saharan Africa ranges from 12.5 to 27.1% in pregnancy and from 10.0 to 34.5% postnatally [1, 22]. A systematic review by Sawyer and colleagues found that rates of depression were 11.3 and 18.3% during ante- and postnatal periods, respectively [18], whereas prevalence rates of antenatal and postnatal anxiety were 14.8 and 14% respectively [18]. Other studies, however, have found even higher rates. In a low-income setting in Cape Town, 39% of pregnant women screened positive on the Edinburgh Postnatal Depression Scale (EPDS: scores ≥14) for depressed mood and 34.7% of postnatal women were diagnosed with depression [23]. In a study conducted in Uganda, 43% of the participants were found to have postpartum depressive symptoms on the EPDS (scores ≥10) [24]. Rates may vary according to whether the tools in these cited studies were screening tools or diagnostic tools or interviews, as well as the use of different cut offs, with the EPDS.

Elevated rates have also been found for anxiety, which has been less widely studied in Africa. In a review of studies from Africa, the antenatal and postnatal anxiety prevalence rates were 14.8 and 14%, respectively [18]. However, the relative prevalence across the perinatal period varies widely by study. With some recent studies reporting a higher prevalence of anxiety in the antenatal period [25, 26] than in the postnatal period [26, 27]. The elevated rates of CPMDs in low-income countries may be attributed to difficult living conditions experienced in these settings. This includes both material and social challenges and exposure to stressful life events. Known risk factors for maternal mental health problems include social factors such as poverty, low education levels; poor social support and relationship factors like marital problems [13, 24]. In addition to stressful life events, cultural traditions and values may also alleviate or exacerbate the development of CPMDs [15, 28]. In some regions, being a single mother or having a baby out of marriage is not socially acceptable and stigma or rejection associated with single motherhood can contribute to the development of symptoms of CPMDs [28, 29]. On the other hand, research suggests that in some cultural settings women may receive extra attention after birth, particularly in the first month. In these situations, a woman is typically attended by female relatives or in-laws. Expecting this support but not receiving it may contribute to the development of CPMDs [15, 28, 29].

Many of the known risk factors are widely prevalent in Rwanda; a small East African country of almost 12 million people that is in the bottom third of the Human Development Index [30]. To our knowledge, there is no published research about CPMDs in Rwanda, but available literature indicates that mental health problems such as anxiety and depression are prevalent in the Rwandan population [31, 32]. However, rates of antenatal and postnatal anxiety and depression may be particularly high in the Rwandan context. This study was therefore aimed at determining the prevalence and associated factors of CPMDs (anxiety and depression) in a selected district hospital of the Eastern Province in Rwanda.

## Methods

### Study design and setting

A descriptive quantitative cross-sectional survey was used to measure the prevalence, and factors associated with, the symptoms of CPMDs in a sample of women receiving regular antenatal care and infant immunization. The study was conducted at 5 of 14 health centres (HCs), which were selected using simple random sampling by picking the number assigned to each health centre from a box, and at their affiliated district hospital in the Eastern Province in Rwanda. The administrative district of the study setting is a primarily rural district with one main town serving as the district capital and has 14 health centres, which function as primary health facilities [33]. This province is one of the most populated but has poorer health indicators as compared to other districts in Rwanda. The Rwandan Ministry of Health’s data suggest that 66.1% of women in the selected district deliver in health facilities (national average = 69.2%, the district is ranked 16th of 30 districts), 97.4% attend at least one antenatal care visit (national average = 98.0%, rank 20 of 30 districts), and 21% attend at least four visits (national average = 35.4%, rank 29 of 30 districts) [34].

### Participants

The study population comprised pregnant women attending antenatal care (ANC) or infant immunization in 5 HCs or the affiliated district hospital. To obtain the required sample, a systematic sampling technique was used to recruit a sample of 165 women into the study. Only women over 15 years of age who were pregnant or had a child up to 1 year of age were included. Women during childbirth or in the first 2 weeks postpartum were excluded because of unstable mood associated with physiological changes in the early weeks postpartum [8].

### Materials

The questionnaire had two parts; the first part asked about the socio-demographic characteristics of participants and factors associated with perinatal depression and anxiety identified in past research. Sociodemographic questions included in this analysis are age (classified as less than 24, 25 to 29, 30 to 34, and 35 and older); marital status (single, married, living together, separated/divorced); education (no formal education, any amount of primary school, any secondary school or tertiary); own and husband’s occupation (employed by other, unemployed, self-employed, no permanent job); number of children born to the mother who are still living (0, 1, 2, 3, 4 or more); pregnancy status (pregnant or not); and whether the child or pregnancy was planned (yes/no). Participants were asked about lifetime exposure to stressful events, such as any form of abuse in childhood, sexual abuse, poverty, loss of their husband or child, intimate partner violence, family mental illness or personal illness. Because of a coding error, it was not possible to determine who experienced more than one of these lifetime stressors so stressful events were coded as none versus at least one. Social factors included loss of nuclear family members (parents and siblings alive, lost a parent, lost a sibling, lost both parents and siblings) and the nature of the relationship with the husband (strong/poor). The full survey in English and Kinyarwanda is available as a Additional file 1.

The second part included screening tools widely used and validated across a range of cultural settings from LMICs including Africa [1, 8, 24, 27, 35, 36]. The Edinburgh Postnatal Depression Scale (EPDS) is composed of 10 questions using a 0 to 3 Likert scale [24, 37]. The maximum score is 30. Research conducted in a low resource setting in Uganda, a setting similar to Rwanda, found possible (as opposed to probable) depression at scores of 10 or greater [24], which is consistent with research in other LMICs [20, 24, 37, 38].. Thus, a score of 10 or greater was considered for possible depression in this study. The Zung Self-rating Anxiety Scale (SAS) consists of 20 items rated on a 1–4 Likert scale [27, 39]. The total SAS raw score ranges from 20 to 80; previous studies in LMICs have used a score below 45 to indicate a normal range of anxiety; 45–59 moderate; 60–74 severe and 75 and over indicates extreme anxiety [27, 39]. These standardized scales used were found to be reliable; the Cronbach’s alpha was 0.89 and 0.87 for EPDS and SAS respectively.

The full questionnaire was translated into Kinyarwanda by a professional translator and a mental health expert to confirm the validity of the translation. It was back-translated to English by three mental health experts, who also confirmed the validity of the translation. The questionnaire was also presented to Rwandan academics and mental health professionals and in other academic forums for feedback concerning the content validity and to determine the clarity and fit with research objectives. A pilot study (*N* = 16, or 10% the size of the main study) tested the feasibility of the study, the instruments and suitability of the research design. A total of 16 participants were examined under the same circumstances as the main study, however, findings were excluded from the main study. Minor adjustments for clarity of wording were made before the main study began.

### Procedures

Ethical clearance letters were obtained and permission from the district hospital management was granted. Data collection was then conducted by a research assistant who was an experienced mental health nurse. The research assistant was introduced to each mother by the nurse or midwife in charge of the services while participants were waiting for their follow-up care or that of their infants. He explained the research to them individually and invited them to participate. All participants received a complete description of the study and signed a written consent form prior to participation.

Regular antenatal visits are held on 1 day each week at each health centre. Likewise, immunization is held on 1 day per week at each health centre. Each health centre was visited twice, once on their antenatal visit day and once in the same week on their immunization day. A systematic sampling technique was used, with every 5th eligible client who presented on that day being admitted into the study [40]. Approximately 15 additional participants were recruited from the paediatric ward of the district hospital by asking women waiting in the ward the age of their children, and again selecting every 5th eligible client to participate.

### Ethical considerations

Ethical approval from the Senate Research Committee of the University of Western Cape/ South Africa (Certificate: 13/8/9) and Kigali Health Institute/ Rwanda Institutional Review Board (Certificate: KHI/IRB/26/2013) was obtained, as well as written permission to collect data from the Director of the hospital. All participants provided written consent. Participants under the age of 18 (the age of majority in Rwanda) signed assent forms and their parents/guardians provided written consent. Participants who were found to have EPDS scores above 10 (possible depression) or who endorsed item 10 (suicidal thoughts), and also participants who had SAS scores above 60 (severe anxiety), were first informed of their scores and then allowed to decide whether or not to seek treatment. If they accepted treatment they were helped to make an appointment with the mental health team after they had completed their medical appointment.

### Data analyses

The Cochran formula for small populations was used to determine the sample required for analysis [41], thus the total number of 165 of the sample population was obtained.

Analyses were conducted using SPSS v. 21 and proceeded through the following steps: 1) Cronbach’s alphas were used to test for reliability on the EPDS and SAS; 2) Categorical variables were created. Age was recategorized into 5-year increments, however, the number of women aged 15 to 19 was too small for meaningful analysis (*n* = 4) and so the youngest two categories were combined into one category of 24 and younger for multivariate analyses. Education originally had categories for both secondary and tertiary education but with only 4 participants reporting any tertiary education, this was combined with the secondary category. EPDS was recategorized into 2 levels based on standard cut off scores for possible depression (10 or greater). SAS was recategorized according to standard cut off scores for probable anxiety (≥ 45); 3). Frequencies were used to describe the categorical risk variables, and means and standard deviations were also calculated for the two clinical dependent variables. 4) Bivariate chi-square tests of independence were used to determine the relationship between all predictor variables with the two categorical psychological symptom variables (i.e., EPDS and SAS). A significant strong relationship was observed for the two dependent variables of depression and anxiety symptomatology (Cramer’s V = .55), suggesting that they may be measuring the same underlying construct but since they were analysed separately this was not an issue for analysis; 5) Separate logistic regression models regressed each individual psychological symptom variable (EPDS and SAS) onto only those risk variables that were significantly associated with them at *p* < 0.05 in the bivariate tests. All predictor variables were entered in the same step. Model fit was assessed using the Hosmer-Lemeshow Goodness of Fit test with a critical value of *p* = 0.05. Odds Ratios are reported for each variable, along with their respective confidence intervals. Interaction terms were not included to test moderation because of the number of variables and the sample size; dividing the sample into those women in the antenatal versus postnatal period resulted in limited power and so multivariate analyses were conducted on the entire sample.

## Results

Univariate analyses were used to summarize data in terms of frequency distributions of the variables under study. In this case, socio-demographic variables,information about husband/ partner (relationship with husband/partner and occupation) as well as other variables such as loss of nuclear family members, number of children, planned pregnancy, and stressful events are described in terms of frequency and distribution. In addition, the distribution of respondents by EPDS and SAS scores and perinatal period is also included.

### Sample and socio-demographic characteristics of participants

The sample comprised of 165 participants from age 15 years and above. Participants’ characteristics are provided in Table 1. About half (51.5%) of the sample were pregnant. The most frequent age category was those aged 25–29 years (38.2%) (see Table 1). Almost half of the respondents were married (44.8%). Most were unemployed (77%) and had a primary school level of education only (60.6%). A total of 96 participants (58.2%) had experienced at least one highly stressful life event. As shown in Table 1, 35.7% of respondents had both parents and siblings alive, but the majority of respondents had lost at least one immediate family member, although the reason for this loss (i.e. illness, accident or violence) was unknown. More than a third (40.6%) reported an unplanned pregnancy. Of those living with husband/partners (86.7%), most participants (46%) reported that their husbands/partners were unemployed. When rating the overall quality of the relationship (strong versus poor), more than a half of participants (69.1%) reported a strong relationship with husband/partner while 30.9% reported a poor relationship.

**Table 1:** Socio-demographic characteristics among women in selected health centres and the affiliated district hospital in Rwanda

Demographic characteristics	Frequency (*n* = 165)	Percent (%)
Age in years		
15–19	9	5.5
20–24	46	27.9
25–29	63	38.2
30–34	26	15.8
35 or over	21	12.7
Marital status		
Single	8	4.8
Married	74	44.8
Living together	69	41.8
Separated/divorce	14	8.5
Level of education		
No education	43	26.1
Primary	100	60.6
Secondary	18	10.9
Tertiary	4	2.4
Participants’occupation		
Employed by other	3	1.8
Unemployed	127	77.0
Self-employed	31	18.8
No permanent job	4	2.4
Participants’ nuclear family		
Both parents/siblings alive	59	35.7
Lost a parent	49	29.7
Lost a sibling	30	18.2
Lost both parents/all siblings	27	16.4
Number of children		
No child	29	17.6
One	43	26.1
Two	39	23.6
Three	20	12.1
Four or more	34	20.6
Planned pregnancy		
Yes	98	59.4
No	67	40,6
Total	165	100

### Prevalence of perinatal depression and anxiety symptoms

Scores on the Edinburgh Post-natal Depression Survey (EPDS) for the entire sample ranged from 0 to 30 (*M* = 10.8, *SD* = 8.13). Women who reported 10 or greater scores on the EPDS were coded as having symptoms of depression. Self-Rating Anxiety (SAS) scores ranged from 23 to 74 (*M* = 42.2, *SD* = 12.31). SAS was coded as “0” for the respondents with normal range anxiety and “1” for those with scores for probable symptoms of anxiety (moderate and severe; SAS > 45). The findings indicate that half (50.3%) had symptoms of depression on the EPDS whereas 37% had scores above the cut-point rates (probable symptoms of anxiety) for SAS.

The proportion of women in the postnatal period with symptoms of depression (*N* = 49, 63.6%) and anxiety (*N* = 37, 48.1%) was higher than the proportion with symptoms of depression (*N* = 32: 37.6%) and anxiety (*N* = 24, 28.2%) in the antenatal period; χ^2^ (1) = 10.92, *p* < 0.00, for depressive symptoms, χ^2^ (1) = 13.13, *p* < 0.00, for anxiety symptoms. For the sample overall, there was a significant relationship between having elevated symptoms of depression and anxiety, with 52 (31.5%) women above the cut-off point on both scales, χ^2^ (2) = 49.67, *p* < 0.00, while 31 (18.8%) reported only elevated symptoms of depressive symptoms and 9 (5.5%) reported only elevated symptoms of anxiety.

### Factors associated with perinatal depression and anxiety symptoms

A binary logistic regression was performed to assess the impact of factors on the likelihood of elevated symptoms of perinatal depression (EPDS > 10). The model contained those independent variables that were found to have significant bivariate relationships with the EPDS: age, the highest level of education experienced, relationship with husband; the number of previous children; and having had stressful life events. The full model containing these predictors was statistically significant, Χ^2^ (8) = 9.44, *p* = 0.31. Participants who reported a good spousal relationship (Odds Ratio: 0.09, C.I. =0.03–0.25) were less likely to have elevated depressive symptoms. Those having their first child were less likely to have depressive symptoms relative to those with 4 or more (Odds Ratio: 0.07, C.I. = 0.01–0.42) (see Table 2).

**Table 2:** Logistic regression predicting the likelihood of symptoms of perinatal depression among women in selected health centres and the affiliated district hospital in Rwanda

Independent variables	B	Wald	df	p	Odds Ratio	95% C.I.		% Screen High EPDS Within Group
						Lower	Upper	
*Age*		7.84	3	0.05				
24 or less	1.06	1.52	1	0.22	2.89	0.53	15.64	50.9
25–29	−0.04	0.00	1	0.96	0.96	0.19	4.74	46.0
30–34	−1.03	1.60	1	0.21	0.36	0.07	1.76	46.2
35 or more (r)					1.00			66.7
*Level of education*		8.74	2	0.01				
No Education	0.69	0.99	1	0.32	2.00	0.51	7.83	76.7
Primary	−0.78	1.69	1	0.19	0.46	0.14	1.49	42.0
Secondary or more (r)					1.00			36.4
Relationship with husband*								
Strong	−2.43	21.16	1	0.00	0.09	0.03	0.25	34.2
Poor(r)					1.00			86.3
Number of children*		10.48	4	0.03				
No Child	−2.10	8.29	1	0.00	0.07	0.01	0.42	20.7
One	−1.32	2.73	1	0.10	0.27	0.06	1.28	51.2
Two	−0.92	1.37	1	0.24	0.40	0.09	1.86	46.2
Three	−0.13	0.03	1	0.87	0.88	0.18	4.26	60.0
Four and more (r)					1.00			73.5
*Stressful life events*								
None	−0.46	1.18	1	0.28	0.63	0.28	1.44	34.8
At least one (r)					1.00			61.5
Constant	3.15	11.12	1	0.00	23.36			

*: Predictors with a *p* < .05, “r”: Reference category, *df* degree of freedom, *p p*-value, *B* Regression coefficients

A logistic regression model was used to determine the extent to which the following variables were associated with elevated levels of symptoms of perinatal anxiety on the SAS: the relationship with husband, planned pregnancy, and the experience of stressful life events. The full model containing the above factors was statistically significant, χ^2^ (8) = 5.73, *p* = 0.68.

The results indicate that, in a multivariate analysis, past exposure to stressful life events was the only significant correlate of symptoms of perinatal anxiety. Respondents who reported not having had any extremely stressful life events were less likely to have anxiety symptoms (Odds Ratio = 0.20, C.I. = 0.09–0.44) than those who had not (see Table 3).

**Table 3:** Logistic regression predicting the likelihood of symptoms of perinatal anxiety among women in selected health centres and the affiliated district hospital in Rwanda

Independent variables	*B*	Wald	*df*	*p*	Odds Ratio	95% C.I.		% Screen High on SAS
						Lower	Upper	
*Relationship with husband*								
Strong	−0.47	1.43	1	0.23	0.62	0.29	1.35	28.9
Poor (r)					1.00			54.9
*Planned pregnancy*								
Unplanned	−0.63	2.76	1	0.10	0.53	0.25	1.12	28.6
Planned (r)					1.00			49.3
*Number of Children*		6.00	4	0.20				
No Child	−1.20	3.35	1	0.07	0.30	0.08	1.09	17.2
One	−0.21	0.16	1	0.69	0.81	0.29	2.28	34.9
Two	−0.48	0.80	1	0.37	0.62	0.21	1.78	30.8
Three	0.44	0.50	1	0.48	1.56	0.46	5.28	55.0
Four or more					1.00			52.9
Stressful life events*								
None	−1.62	15.33	1	0.00	0.20	0.09	0.44	15.9
At least one (r)					1.00			52.1
Constant	0.99	5.13	1	0.02	2.70			

*: Predictors with a *p* < .05, “r”: Reference category, *df* degree of freedom, *p p*-value, *B* regression coefficient

## Discussion

The findings of this study suggest that symptoms of depression and anxiety were relatively high among women using health clinics for basic antenatal care and infant immunization. Although we found relatively high rates of perinatal depression symptoms, they are comparable to other studies using the same cut-off of 10 for possible depression on the EPDS. A study conducted in a rural district of the neighbouring country, Uganda, also reported that elevated symptoms of postnatal depression (43%) [24] and high rates have also been observed in some studies in South Africa (e.g., 34.7%) [23].

Symptoms of perinatal depression were higher postnatally than antenatally. Some African studies have found higher rates of symptoms of antenatal depression than postnatal [42, 43]. However, a recent review of research in low and middle-income countries found antenatal rates to be lower than postnatal rates [17], a pattern also obtained in high-income countries [44]. The lower prevalence of symptoms of antenatal depression should not be neglected, however, as antenatal depression is known to negatively impact on the uptake of antenatal care, foetal and obstetric outcomes, and is a strong predictor of postnatal depression [43].

The elevated rates of anxiety symptoms are also of concern. There is a growing body of research that suggests that perinatal anxiety is at least as disruptive as depression and possibly more prevalent [45], with potential adverse consequences for the mother’s health, the early infant relationship and the child’s health and development [18].

### The factors associated with symptoms of perinatal depression and anxiety

The pervasive impact of both the immediate and larger social context on rates of symptoms of perinatal depression is apparent in the variables associated with women’s perinatal mental health. Marital status was not significantly associated with symptoms of perinatal anxiety in the bivariate analysis but was associated with symptoms of perinatal depression. The latter finding is consistent with recent studies in Africa [18, 43]. For those who had a partner (common-law or married), the relationship quality was found to be a strong correlate of symptoms of both perinatal depression and anxiety. Marital problems and lack of emotional and practical support from spouses have been found to be important in the development of CPMD in several other studies in Africa [18, 23, 24].

The absence of supportive relationships, in general, is particularly salient in Rwanda, a country that has seen a profound disruption of community and family relationships as a result of the 1994 genocide against Tutsi [46]. Many Rwandans lost family members to the conflict and while Rwanda has a history of collective support, rebuilding trust and supports following the genocide has been a long process [47]. This renders new mothers more vulnerable to perinatal depression, which in turn affects the well-being of not only the women themselves but also the cognitive and emotional development of their children [5, 13]. This study did not distinguish between losses that occurred during the genocide and those that occurred at other times; it would be helpful to know when and how these losses happened. Losses due to the genocide would be associated with an intensely traumatic event, which may not be the case for those due to illness or accidents, would carry a very different meaning and be understood differently, and would have happened 20 years in the past. As such, their impact may be very different from those losses sustained through other experiences, something which would benefit from further study. Another interesting area for further research would be to explore whether the impact of a poor relationship with a partner may be mitigated by other sources of support for women in these environments (e.g., peer support from neighbours or friends). Fisher and colleagues highlighted that nurturing and confiding intimate relationships exercise a protective influence on maternal mental health [8]. Building community in this way can also create better social environments and opportunities for the community as a whole, reinforcing how the well-being of mothers and their children is connected to that of their broader social environment, a point that is core to the Bronfenbrenner’s social ecological model of development [48].

What was perhaps surprising was how few other variables of the larger social context were associated with symptoms of perinatal depression and anxiety. There was a difference between mothers of 4 or more children and those with no children, a pattern that has been observed elsewhere [25, 49]. This may reflect the impact that having to manage a large family in a region with such limited resources can have on women’s mental health. It should be noted, however, that the presence of other children in the household was not accounted for, including children of the woman’s spouse, and thus the impact of the number of children may be understated here.

Higher socioeconomic and education levels have been found to be protective factors for mood and anxiety disorders in the perinatal period [17, 25] but while we found a link between the level of education and symptoms of perinatal depression using simple bivariate analyses, this effect disappeared in a multivariate analysis. For perinatal anxiety symptoms, there was no association with employment or education. However, there may have been a floor effect in this study, as most mothers (77%) were unemployed, as were their spouses.

Likewise, in multivariate analyses, past stressful life events did not predict mothers’ symptoms of perinatal depression despite their importance in other research [17, 18, 24, 25, 38, 50, 51]. However, exposure to extremely stressful past events was the only predictor of perinatal anxiety. It is important to note that the entire population underwent a traumatic event in recent history, the 1994 genocide against the Tutsi. It is possible that, in this context, the questions asked about the loss of family members and personal stressful life events did not adequately distinguish between the kinds of past trauma and loss women may have experienced in the context of predicting depression, or that there is a baseline of traumatic experience that all have already exceeded by virtue of living in a post-genocide society.

### Limitations to the study

Our study’s relatively small sample size may have lacked the power to identify more modest but important relationships between the variables under study, or to explore moderating relationships, and also limits its generalizability to Rwanda as a whole. There is also some concern regarding selection biases, given that it was a clinic-based study, and only in one district. As such it may underestimate the prevalence of symptoms of perinatal depression and anxiety because the mothers who did not attend their appointment time within the period of the study were not sampled and this may have precluded the most vulnerable mothers from the study. Women who may be suffering from symptoms of CPMD may lack the motivation or ability to visit a health facility, not only for themselves but also for the care of their infants. Moreover, women’s social vulnerability differs from province to province in Rwanda, and thus these women may not be representative of the wider population. Thus, there is a need for community-based research in a wider range of provinces and districts to assess common perinatal mental disorders.

All variables were self-report and it is possible that women were not comfortable disclosing information about their own levels of distress as a result of concerns about social desirability and stigma or their spousal relationships. Nonetheless, it would have been informative to have more information about the level of conflict in women’s relationships (e.g., violence or alcohol abuse). It would also have been informative to know more about the number of stressful events participants had been exposed to in their lifetime but this was not possible due to the way the data were collected. Knowing about whether stressful life events were experienced currently or in the past would also have been informative. The study is also limited by its cross-sectional design. Caution must be taken in assuming causal relationships between these variables. Longitudinal research is needed to explore whether causal relationships exist between these variables. Additionally, researchers only used screening tools, and so these findings do not reveal diagnostic rates of depression and anxiety diagnoses. Finally, research validating the EPDS or SAS to be reliable instruments in the Rwandan context is needed.

## Conclusions

The high rates of symptoms of perinatal depression and anxiety indicate that it is imperative to include screening for CPMDs in order to improve detection and referral for interventions. Moreover, public education about CPMDs and training for all providers of services for pregnant and postpartum women is essential to increase awareness and early detection as well as to promote access to care.

This research raises important questions about associated factors of mental health in social contexts with limited social and material resources. The results here suggest that women in highly vulnerable environments may be particularly dependent on support available from their immediate social networks, which likely includes both material and social support, and particularly the presence of a good relationship with their husband/partner. Prevention strategies should focus on addressing the social conditions of women in the perinatal period. Health authorities and policy makers should consider the integration of maternal mental health care into maternal health. Thus, health care providers concerned should receive continuous professional development and in-service training enabling them recognizing risk factors, detecting and intervening early for women with CPMDs.

## Supplementary information

**Additional file 1.** Reviewer reports.

## Data Availability

The datasets used and analysed during the current study are available from the corresponding author on reasonable request.

## Abbreviations

CPMD: Common perinatal mental disorders
EPDS: Edinburgh post natal depression scale
SAS: Self-rating anxiety scale
