# Supplementary material for: A cross-sectional study of the prevalence and factors associated with symptoms of perinatal depression and anxiety in Rwanda
Source: BMC Pregnancy Childbirth. 2020 Jan 31;20:68. doi: 10.1186/s12884-020-2747-z (PMC6995211; doi:10.1186/s12884-020-2747-z)
Supplement: Supplementary file 1 — Additional file 1. Reviewer reports. [file 12884_2020_2747_MOESM1_ESM.docx]

Reviewer reports:

Roseli M Y Nomura, Ph.D., M.D. (Reviewer 3):

| Abstract: Please give the criteria for inclusion in the methods describing what is the perinatal period. | Information provided see abstract |
| --- | --- |
| Results: correct the results presented here as pointed in page 13, lines 32 to 40, | Corrected |
| The introduction is long, should be reduced. The number of references is excessive. Please shorten the introduction and reduce the number of references. | Introduction reduced and references |
| It is necessary to present clearly the purpose of the study at the end of the last paragraph of the introduction. | The purpose is added see page 6 |
| Methods:  Page 7, Line 43: What's the meaning of "... identified in past research." Were participants selected in another research? | These are factors associated with perinatal depression and anxiety identified in the past research not participants. |
| Page 9, line 29: The authors inform verbal consent. However, in page 10, line 1, it is written that all provided written consent. Please, explain or correct. | Corrected see page 9 |
| Results  Page 11, line 31: As reliability results were not the aim of this study, authors should include these results (cronbach's alpha) in the methods (Page 8, Line 51). | Cronbach's alpha put in the methods see page 8 |
| Page 12, line 17 and line 22: present only %, not numbers. | Correction made see page 11 and 12 |
| Discussion: OK | Thank you |
| Conclusion: is too long. It should be shortened. | Conclusion shortened |
| Table caption are insufficient to accurately describe table contents. Use more complete titles, describing at least the location and the population analyzed. | Titles edited |
